# Knowledge engagement in gene drive research for malaria control

**DOI:** 10.1371/journal.pntd.0007233

**Published:** 2019-04-25

**Authors:** Sarah Hartley, Delphine Thizy, Katie Ledingham, Mamadou Coulibaly, Abdoulaye Diabaté, Bakara Dicko, Samba Diop, Jonathan Kayondo, Annet Namukwaya, Barry Nourou, Léa Paré Toé

**Affiliations:** 1 Department of Science, Innovation, Technology and Entrepreneurship, University of Exeter, Exeter, United Kingdom; 2 Department of Life Sciences, Imperial College London, London, United Kingdom; 3 Malaria Research and Training Center, University of Sciences, Techniques and Technologies of Bamako, Bamako, Mali; 4 BioMedical Département, Institut de Recherche en Sciences de la Santé, Bobo-Dioulasso, Burkina Faso; 5 Department of Entomology, Uganda Virus Research Institute, Entebbe, Uganda; 6 Malaria and Neglected Tropical Diseases, Institut de Recherche en Sciences de la Santé, Bobo-Dioulasso, Burkina Faso; Universita degli Studi di Pavia, ITALY

## Introduction: Development of gene drive mosquitoes

Gene drive mosquitoes are a novel approach to vector control being developed to help tackle malaria. A gene drive increases the frequency of a desired gene and its phenotypic effect into a mosquito population through reproduction in relatively few generations [1]. Combining gene drive with the precision of gene editing, scientists are able to modify the *Anopheles* mosquito genome and push modifications through natural vector populations. Population-suppression drives restrict the population of *Anopheles* mosquitoes through the spread of recessive lethal and sterility genes or by biasing the sex ratio [2]. Population-replacement drives interfere with the ability of the *Anopheles* mosquito to transmit the *Plasmodium* parasite [3]. The potential benefits of gene drive may be significant, particularly in sub-Saharan Africa, where malaria is endemic and increasing insecticide and drug resistance threaten health gains made in this area [4].

Scientists and funding bodies have made repeated calls for public engagement in gene drive [5, 6]. In 2016, the National Academies of Sciences, Engineering, and Medicine (NASEM) published its report, *Gene Drives on the Horizon*: *Advancing Science*, *Navigating Uncertainty*, *and Aligning Research with Public Values* [7]. The report identified public engagement as a key area of responsible science, defining engagement as “seeking and facilitating the sharing and exchange of knowledge, perspectives, and preferences between or among groups who often have differences in expertise, power, and values” [7]. Researchers are asked to participate in two-way engagement with publics (defined as stakeholders, communities, and the public) to allow their knowledge to contribute to technology development and align the technology with public values. In this viewpoint, we share our initial research findings in this area and propose a conceptual tool that contributes to the debate at this critical juncture.

## Engaging knowledge rather than publics?

The NASEM report reflects a shift in thinking about public engagement from a knowledge-deficit to a co-development approach. Traditional knowledge-deficit approaches are often based on scientists’ perception that publics do not understand the technology or will fear new biotechnologies based on experiences with genetically modified crops. Knowledge-deficit approaches tend to result in top-down activities designed to educate publics about the benefits of the technology in order to secure acceptance or consent for a field trial. In order to achieve the co-development approach that NASEM outlines, knowledge engagement must be disentangled from knowledge-deficit types of public engagement and allow for the collaborative reconfiguration of gene drive technology design and implementation with publics.

In response to NASEM, the former minister of health in the Republic of Namibia, Richard Kamwi, emphasizes the importance of knowledge engagement for African expert publics and calls for the early involvement of African scientists and disease control experts in gene drive research. Kamwi argues that African expertise in local epidemiology and entomology “will be vital to determining how gene drives may one day be applied to mosquito control” [8]. Kamwi’s assertion builds on the observation of Hassan Mshinda, director general of the Tanzania Commission for Science and Technology, that there is an urgent need for malaria-afflicted nations to interact with overseas collaborators as “equal partners” [9]. Mshinda and colleagues emphasize that, “unlike cutting-edge molecular biology, semi-field ecological studies and open-field research can be undertaken in any African setting, and constitute an immediate opportunity for malaria-afflicted nations to regain their role as stakeholders, decision-makers and eventual owners of this technology” [9].

We are a collaboration of social scientists, natural scientists, humanities researchers, and engagement practitioners working at the intersection of two separate gene drive projects: Target Malaria, a not-for-profit research consortium, and a British Academy–funded project on co-development in the United Kingdom and Mali. We are responding to the call of WHO and other bodies to share experiences about engagement in gene drive [7, 10]. Our British Academy research explores meanings and practices of co-development. Co-development is a term mobilized by both UK and African researchers. For example, the 2018 African Union report *Gene Drives for Malaria Control and Elimination in Africa* argues for a model of co-development that engages African experts, communities, stakeholders, and publics, encouraging ownership of the technology in user communities [11].

Target Malaria emphasizes that engaging diverse types of knowledge is essential for achieving its overarching goal of co-developing the technology with African partners. In 2017, Target Malaria held an internal workshop to explore its vision and values. One of the key findings to emerge from this workshop and from our ongoing research was that the adoption of a “knowledge engagement” lens (rather than a “public engagement” lens) allowed Target Malaria to critically reflect on the mechanisms in place to allow diverse types of knowledge to shape research trajectories. Tracing and measuring knowledge flows from scientists to publics is relatively well developed [12]; however, tools to track knowledge flows from publics to scientists are less developed, particularly if the goal is to examine the impact of knowledge on the technology development process.

**Fig 1:**
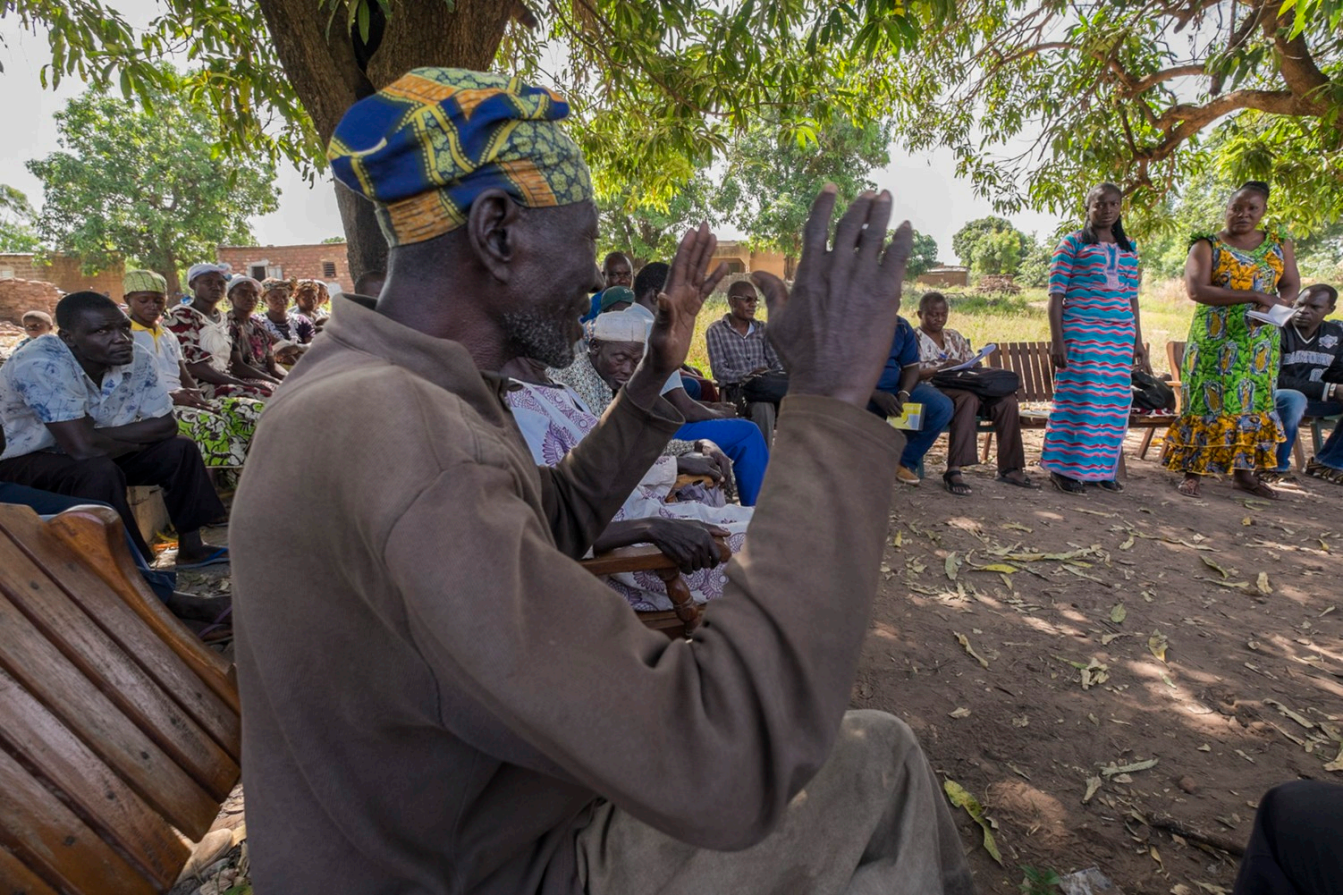
Listening and talking in Burkina Faso. 2015 Target Malaria—Imperial College London. *Photographer*: *Axel Fassio*.

Target Malaria is not alone in its commitment to reimagining engagement. Other strategies for vector control have made important strides toward socially and culturally sensitive engagement with communities impacted by field trials [13, 14]. However, these approaches do not go far enough. There is little recognition that communities possess knowledge that could usefully shape technology design. Difficulties have been reported elsewhere in envisaging the engagement scenarios that might enable groups to have an impact on genomic modification technology [15]. NASEM describes engagement as “listening as well as talking.” This description requires that technology developers receive knowledge from multiple publics and integrate that knowledge into gene drive design and development [7] (Fig 1). In Mali, a collaborating research site of Target Malaria and the British Academy project, terms such as “gene” do not translate readily into local dialects, providing opportunities for technology developers to listen and learn as well as talk and disseminate.

## Rationales for engagement

A significant finding to emerge from our preliminary research is that there is an urgent need for greater conceptual and empirical delineation of how and why knowledge engagement matters and how to conduct it. The majority of governance documents on gene drive in global health focus on community, stakeholder, and public engagement driven by normative and instrumental rationales [5, 7, 10, 11, 16]. For example, *Pathway to the Deployment of Gene Drive for Malaria Control in Sub-Saharan Africa* describes engagement as “essential to meeting ethical obligations of informed consent, building trust, and gaining acceptance of the research” [16]. Elsewhere in the document, reference is made to substantive motivations for engagement—for example, in engaging communities to “understand what characteristics would make the product attractive from their perspective” [16]. However, there is frequent slippage to a reductive rendering of engagement as the right thing to do or as a way to secure public acceptance. There is also minimal explication surrounding how engagement might be practiced with multiple publics in richer ways. Although the ethical motivations for engagement are important, our African partners emphasize substantive motivations for engagement in which specific knowledge can contribute to the robustness of gene drive. Knowledge engagement is a tool for public engagement driven by substantive motivations that recognizes that diverse types of knowledge can craft better technologies and strategies for disease control.

## Future directions

Although the gene drive research community is fully committed to diverse engagement activities and technology co-development, it is not yet clear how knowledge is used by researchers who have the ability to shape the technology’s trajectory and whether research teams are open to a range of eventualities (such as social, political, and ecological challenges; plausibility under real-world conditions; and changing definitions of the problem). Our continuing empirical research is underway in the UK and Mali to explore the impact of Malian knowledge on decisions in the technology development process.

Currently in Ghana, Target Malaria is opening up the research process through the development of an ecological observatory where knowledge from and questions raised by diverse publics surrounding the ecological implications of the suppression of *Anopheles gambiae* are shaping research programs. In Uganda, another of Target Malaria’s research sites, fish are an integral component of local diets. Target Malaria is here collaborating with local publics to define socially and culturally relevant environmental protection goals. These examples provide key lenses through which to develop greater explication surrounding how diverse types of knowledge might shape the trajectories of gene drive.

In the meantime, there is much that Target Malaria and gene drive developers can learn from previous scholarship that demonstrates that diversifying the kind of knowledge shaping a technology may increase its efficacy and social robustness [17]. We hope that by highlighting the role of knowledge engagement (rather than “public” or “community” engagement) in the co-development of gene drive mosquitoes, we can help to navigate the enormous challenge of engagement in high-technology research developed for use in low-income countries. Knowledge engagement is an innovative dimension of public engagement that may help actors resist slippage toward deficit approaches that mimic outreach and education rather than listening and sharing with publics. If there is a genuine desire for co-development of technology in global health, we need to move beyond thinking about public engagement in normative and instrumental ways and toward investigating the factors that explain how diverse types of knowledge shape the design, development, and implementation of gene drive mosquitoes.
